# How methyl–sugar interactions determine DNA structure and flexibility

**DOI:** 10.1093/nar/gky1237

**Published:** 2018-12-12

**Authors:** Korbinian Liebl, Martin Zacharias

**Affiliations:** Physics Department T38, Technical University of Munich, James-Franck-Str. 1, 85748 Garching, Germany

## Abstract

The sequence dependent structure and flexibility of the DNA double helix is of key importance for gene expression and DNA packing and it can be modulated by DNA modifications. The presence of a C5′-methyl group in thymine or the frequent C5′-methylated-cytosine affects the DNA fine structure, however, the underlying mechanism and steric origins have remained largely unexplained. Employing Molecular Dynamics free energy simulations that allow switching on or off interactions with the methyl groups in several DNA sequences, we systematically identified the physical origin of the coupling between methyl groups and DNA backbone fine structure. Whereas methyl-solvent and methyl–nucleobase interactions were found to be of minor importance, the methyl group interaction with the 5′ neighboring sugar was identified as main cause for influencing the population of backbone substates. The sterical methyl sugar clash prevents the formation of unconventional stabilizing hydrogen bonds between nucleobase and backbone. The technique was also used to study the contribution of methyl groups to DNA flexibility and served to explain why the presence of methyl sugar clashes in thymine and methyl-cytosine can result in an overall local increase of DNA flexibility.

## INTRODUCTION

**Figure 1.**
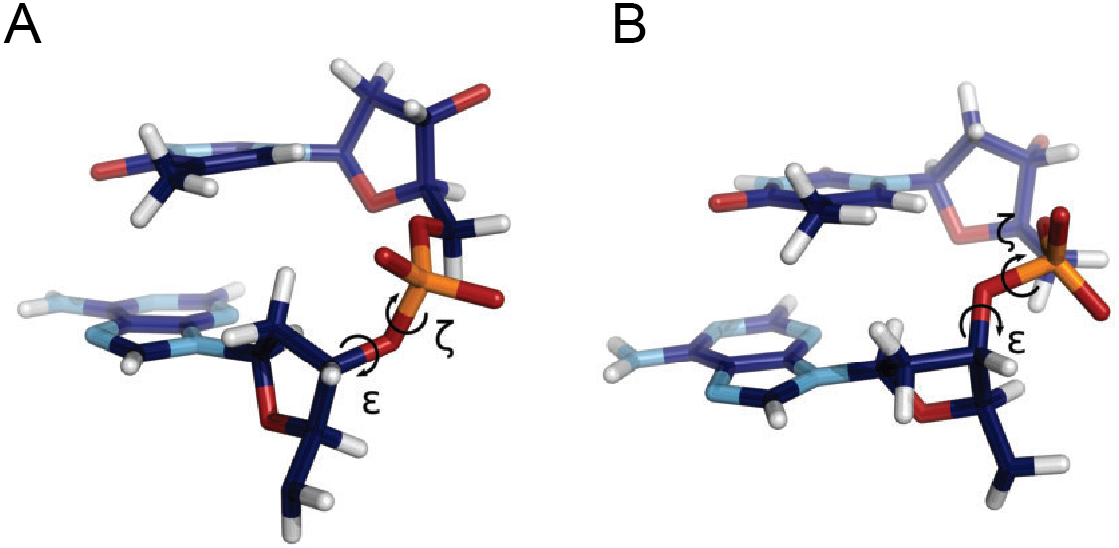
ApT base-pair step with a BI backbone conformation (**A**) and a BII conformation (**B**).

The structure and flexibility of double-stranded DNA and the binding of proteins is influenced by the nucleic acid backbone structure (1–3). One of the most prominent conformational polymorphism in DNA is due to two different combinations of the ε and ζ dihedral angles in nucleotides adopting either the canonical BI (ε/ζ in the *trans*/ *gauche*-) or BII configuration (ε/ζ in the *gauche*-/*trans*, Figure 1). These substates also contribute to the bimodal distribution of a base-pair step’s twist, are significantly affected by mechanical stress and are coupled to the dimensions of minor and major groove (4–7). The distribution of BI/BII substates in DNA can influence protein binding and may also change upon protein binding (8–12).

In previous Molecular Dynamics (MD) simulation and NMR based studies, the sequence dependent population of BI/BII states and their impact on DNA’s structure has been extensively investigated (13–18). Recently, the sequence dependence of BI/BII states was systematically analyzed using trajectories obtained by the Ascona B-DNA consortium (ABC) based on a database of extensive MD simulations of DNA oligomers containing all 136 distinct tetranucleotide sequences (15,19). It was found that the sequence dependent formation of unconventional hydrogen bonds between base and backbone atoms plays a key role in stabilizing the BII substate (a C8-H8..O3′, between purine base and backbone in case of RpR and YpR steps; and a C6-H6..O3′ contact in RpY and YpY steps(15,19)). The formation of a (base) C-H..O3′ (sugar) hydrogen bond contact perfectly correlated with the formation and percentage of BII states at dinucleotide steps in DNA. A hierarchy of bond strengths can be established from the populations observed in simulations (for each dinucleotide step and sequence context) that can be used to interpret the observed sequence-dependent BI/BII propensities (19–21). However, in principle, all purines and pyrimidines in DNA can form such contacts based on the same sterical and geometrical reasons, hence, the observed correlation does not offer a direct sterical explanation.

The presence of a methyl group in case of thymine and also the C5-methylation of cytosine has an influence on the DNA backbone structure and on the occurrence of BII states. The impact of methyl groups on DNA’s structure has been addressed in several previous studies (22–24), however, the sterical mechanism by which methyl compounds influence the DNA backbone has still remained enigmatic. Methyl groups are directed towards the major groove in case of thymine and C5-methylated cytosine and are hydrophobic. Consequently, one might expect the altered hydration pattern in the major groove to be a central feature of DNA methylation (25,26). Furthermore, it has also been stated that for C5-methylated cytosine BI states are stabilized by a water molecule bridging between the methyl-carbon and a phosphate bound oxygen (24). The hypothesis has, however, been revised in a follow-up study by Wibowo *et al.* (27) that rather claims that methyl induced BI stabilization is due to an increased mean water residence time around base atoms.

The repulsion between methyl groups and the sugar ring of the 5′-neighboring base might stabilize the population of BI states (28). Indeed, MD simulations by Peguero-Tejada and coworkers (23) indicate higher populations of BI states for DNA containing thymines compared to analogs with uracil instead of thymine.

Finally, BI states are possibly stabilized through methyl-π stacking with adjacent bases. Interactions between the thymine-methyl group and a 5′-neighboring base are attractive and might have important implications on the deformability of DNA (29–31).

In order to investigate the molecular mechanism and sterical origins on how the presence of a methyl group in thymine and in C5-methylated cytosine influences the ratio of BI/BII states and DNA deformability we performed comparative MD simulations including or omitting non-bonded interactions between nucleobase methyl groups and other parts of the DNA and solvent. The simulations reveal that methyl–π stacking and interaction of methyl groups with solvent have negligible impact on DNA’s backbone structure. However, turning-off interactions between methyl groups and the sugar C2′ atom of the 5′-neighboring nucleotide and its hydrogen atoms drastically increases the population of BII states. Besides influencing the BI/BII ratio methyl groups may also influence the intrinsic flexibility of DNA (32). Using the same technique we also examined how methyl–sugar clashes influence structure and flexibility of the base pairs. Methyl–sugar clashes are predicted to increase the intrinsic bending but significantly reduce DNA’s local stiffness with the bending stiffness reduced by up to ∼40% and the stretching stiffness to ∼35%. Most striking are the calculated effects on the twist-stiffness: For G–T and C–T steps, these clashes reduce the twist stiffness by ∼50–60%. The simulations allow us to explain the observed effects based on sterical effects of the methyl groups. Given the substantial contribution of methyl–sugar clashes to DNA’s stiffness, we suppose that this atomistic effect is of significant biological relevance, e.g. for the regulation of gene expression.

## MATERIALS AND METHODS

### Force field variation

In total, seven different sequences of 15 base pair (bp) DNA duplexes have been studied. Starting structures were generated using the nab module of the Amber16 package (33).

The xleap module of Amber16 was used to generate parameter topology files based on the parmbsc1 force field (34) and the DNA structures were solvated in explicit solvent (TIP3P water model (35)) within a rectangular box and a minimum distance of 10 $\mathring{{\rm A}}$ between DNA and box boundaries. Potassium ions were added in order to neutralize the systems. For the base-atoms of C5-methylated cytosine we used the parameters by Rauch *et al.* (24). These parameters have served as extension to the parm99 force-field. However, updates to the parm99 force-field (e.g. parmbsc1) have addressed torsional backbone angles and not nonbonded parameters of the base-atoms. Hence, using the parameters of Rauch *et al.* (24) in combination with a nucleic backbone according to the parmbsc1 force field gives best compatibility with the force field description of all other nucleotides. Classical MD force-fields have the form:

(1) \begin{eqnarray*} E &=& {\sum_{bonds}} {k_{b}} (r - r_{0})^{2} + \sum _{angles} k_{\theta } (\theta - \theta _{0})^{2} + \nonumber \\ &&+ \sum _{dihedrals} V_{n} [1 + \mathrm{cos}(n \phi - \gamma )] + \sum _{i=1}^{N-1}\sum _{j=i+1}^{N} \frac{q_{i}q_{j}}{r_{ij}} + \nonumber \\ &&+ \epsilon _{ij} \left[\left(\frac{R_{min,ij}}{r_{ij}}\right)^{12} - 2\left(\frac{R_{min,ij}}{r_{ij}}\right)^{6}\right], \end{eqnarray*}

where the last two terms describe the interactions between all non-bonded atoms and are composed of Coulomb and van-der-Waals contributions that allow us to specifically modify interactions between pairs of atoms.

Besides regular parameter topologies, we also prepared topology files with modified force-field descriptions. This includes the modification of the non-bonded interactions between methyl groups and specific partner groups using the parmed module of Amber16. In order to eliminate specific non-bonded interactions, the partial charge on each atom of the methyl groups (C7, H71, H72, H73) was removed and partial charges on adjacent atoms were redistributed in accordance to the charge distribution in the de-methylated analogs. In addition, the pair-wise van-der-Waals parameters between methyl group and defined partner group were set to zero. Hence, for each sequence three additional parameter topologies were generated, neglecting either:

- interactions between methyl groups and all water molecules
- or interactions between methyl groups and the C2′ atom and its hydrogens of the 5′-neighboring sugars
- or interactions between methyl groups and 5′ neighbored bases.

### Simulation setup and equilibration

All simulation systems were first energy minimized with the steepest descent method in 2500 steps by using the sander module of the Amber16 package. All subsequent MD simulations were performed with the pmemd.cuda module of the Amber16 package. Initially, the systems were heated up to 300 K in three stages (in 100 K steps). Each stage was simulated for 100 ps and included positional restraints on all non-hydrogen atoms with respect to the B-DNA starting conformation. Subsequently, positional restraints were gradually reduced from 25 kcal/(mol $\mathring{{\rm A}}^2$) to 0.5 kcal/(mol $\mathring{{\rm A}}^2$) in five consecutive simulations at 300 K and at constant pressure of 1 bar (weak coupling with a time constant of 5 ps). The equilibration phase was completed by a 2 ns simulation, during which only the first two base pairs were positionally restrained with a small force constant of 0.1 kcal/(mol $\mathring{{\rm A}}^2$) which avoids overall rotation of the DNA in the simulation box. The equilibrated structures served as input for the production runs for each force field topology, during which we kept the soft restraints on the terminal bases-pairs. Data gathering simulations were carried out for 900-4000 ns. Coordinates were written out every 5000 steps. Using hydrogen-mass-repartitioning allowed us to use a time step of 4 fs. Details on the calculation of free energy profiles, calculations of errors and convergence and hydrogen bonding as well as conformational deformabilities are given in Supporting Information (Sections 1–5).

## RESULTS AND DISCUSSION

### DNA backbone substates

MD simulations were performed on seven DNA duplexes with different central sequences (Table 1) to record structural fluctuations including frequent transitions between DNA backbone substates to sample substate populations. In a given dinucleotide step, a DNA backbone strand can either adopt BI or BII configurations that are determined by the ε and ζ dihedral angles (Figure 1):

(2) \begin{equation*} \mathrm{BI} : \epsilon - \zeta < 0 \quad , \quad \mathrm{BII}: \epsilon -\zeta >0 \end{equation*}

**Table 1.** Sequences of the studied DNA duplexes

DNA sequence	abbrev.	P(BI) [%]
5′-CGCGC**ATATA**CGCGC-3′	AT	83.6
5′-CGCGC**AUAUA**CGCGC-3′	AU	75.5
5′-CGCGC**GCGCG**CGCGC-3′	CG	73.4
5′-CGCGCGC*GC*GCGCGC-3′	C*G	78.8
5′-CGCGC**AAAAA**CGCGC-3′	AA	88.3
5′-GCGCC**TCTCT**GCGCG-3′	CT	77.2
5′-GCGCG**TGTGT**GCGCG-3′	GT	76.4

C* denotes C5-methylated cytosine (on both strands).

**Figure 2.**
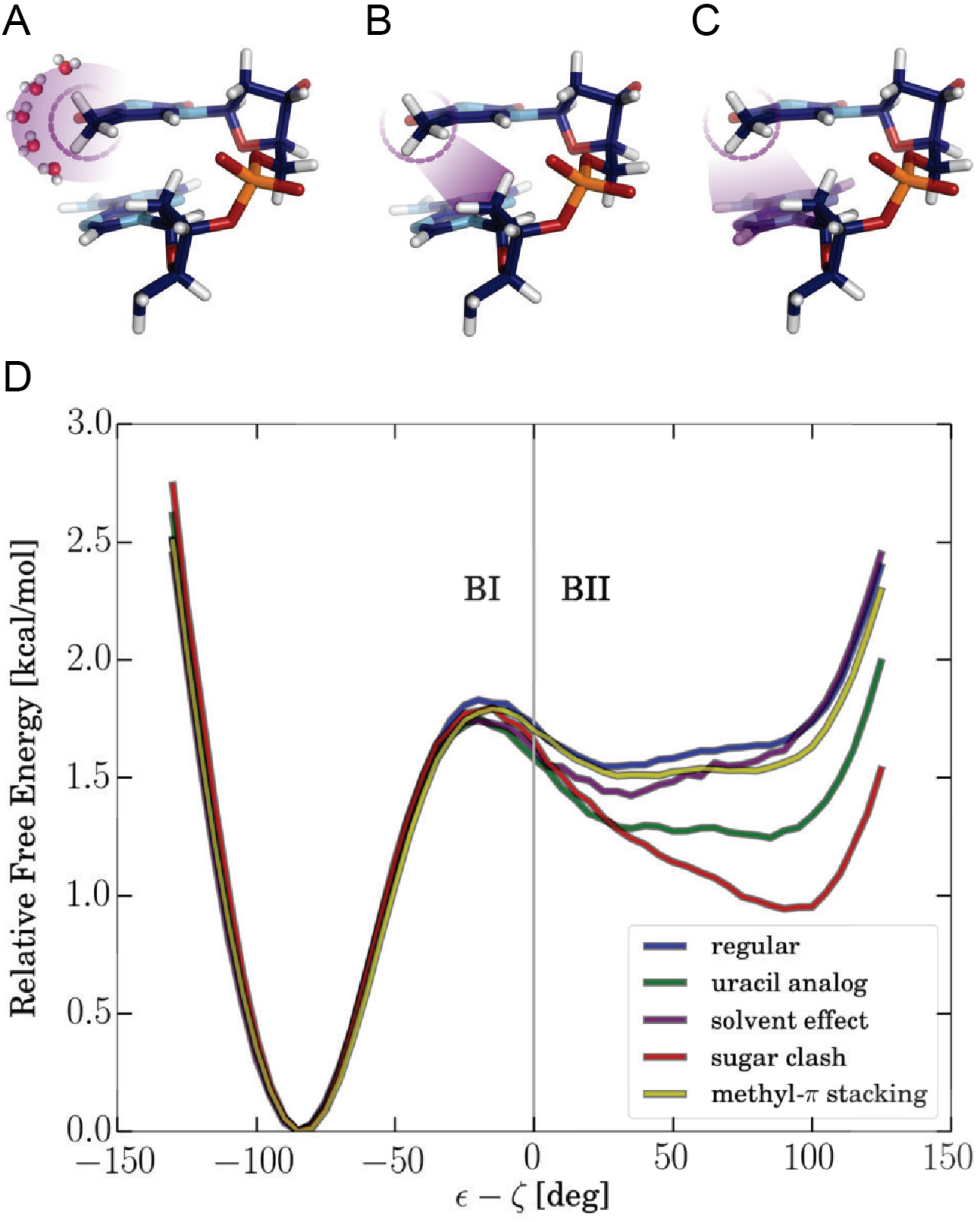
Modulation of the BI-BII free energy profile at central ApT steps by force field modifications. (**A**–**C**) Illustration of specific force field modifications for specifically omitting methyl-solvent interactions (in A), steric clashes between methyl group and 5′-neighboring C2′-sugar atoms (and connected hydrogens, in B) and turning off methyl group interactions with neighboring base atoms (omitting methyl–π stacking interactions, illustrated in C). (**D**) Calculated free energy profiles along the ε–ζ coordinate for the AT-case including all interactions or omitting interactions as indicated in panels A–C (indicated as different line colors or grey scales in the figure panel). For comparison the free energy profile for the AU case is also shown (green curve). The free energies along the ε–ζ coordinate were calculated by Boltzmann-Inversion *F* = −*k*_B_*T* · ln(*p*) and represent the average over all dinucleotide steps in the central segment of the AT (or AU) sequence. The same data (enlarged) is shown in Supplementary Information Figure S1 including error bars.

Transitions between BI and BII substates occur rapidly relative to the total length of the simulations. Hence, it is possible to directly extract probability distributions along the ε − ζ coordinate and to extract associated free energies on the time scale of 900–4000 ns used in data gathering simulations (Figure 2 and Supplementary Information, Section Convergence, Figures S1–S6).

The calculated populations (probabilities) for the canonical BI states for all investigated dinucleotide steps (Table 1) emphasize two findings: First, sequences consisting only of central A–T base pairs (AT,AA) show a higher population of BI states (by ∼10–15%) than other sequences. Second, ‘elimination' of a methyl group (by replacing central thymines by uracil or C5-methylated cytosine by cytosine) results in a lower occupation of BI states (by ∼5–8%, Table 1). Thus, the MD simulations indicate that the presence of methyl groups at the C5 of pyrimidines in DNA stabilizes the BI backbone states. Similar trends have been observed in other studies (23).

In order to understand the molecular mechanism of this stabilizing effect we performed MD simulations of the AT case (central ATATA sequence, Table 1) by turning off non-bonded interactions between the C5-methyl groups and selected subsets of atoms in the system. During the simulations either all interactions of the C5-methyl group were turned off (representing an AU step) or only with the solvent, with the 5′ neighboring base or with the C2′ atom (and its hydrogens) of the 5′ sugar. The associated free energies along the ε–ζ coordinate were calculated by Boltzmann inversion of the sampled probability distributions and represent the average over all steps in the central DNA sequence (steps 6–9). Comparison of the AT versus AU cases indicates a negligible impact of the methyl group on the shape of the free energy curve in the BI subspace. However, the BII regime is of significantly lower free energy resulting in an increased BII population. The exclusion of all interactions between methyl groups and water molecules (Figure 2, purple curve) as well as the exclusion of all interactions between methyl groups and the 5′-neighboring bases (Figure 2, yellow curve) also shows much smaller deviations from the regular case (Figure 2, blue curve). However, excluding interactions between C5-methyl groups and the C2′ atom (and its hydrogens) of the 5′-neighboring sugar results in a large drop of the free energy in the BII regime (Figure 2, red curve). Hence, neither methyl-base stacking nor interactions with the solvent are decisive but the steric clashes between C5-methyl group and 5′-sugar are the main cause of the BI stabilization by the C5-methyl group. It is interesting to note that eliminating only the electrostatic interactions of the methyl group with specific groups had only a small impact on the population of substates but it is dominated by the change in sterical van der Waals interactions (see Supplementary Information Figures S6–S11).

### Why methyl–sugar clashes trigger BI promiscuity

**Figure 3.**
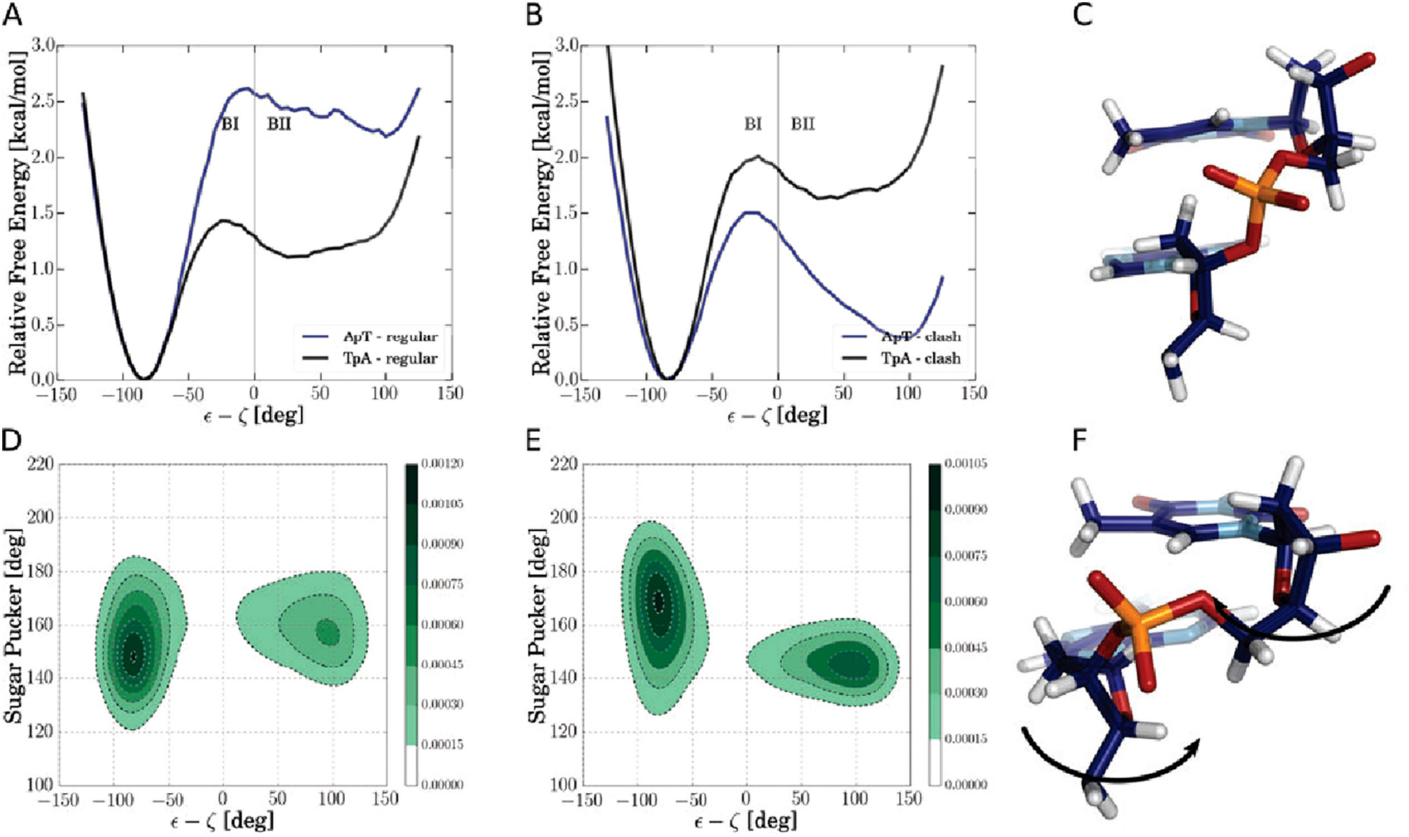
Calculated free energy versus ε–ζ coordinate for single base-pair steps in the AT-case. (**A**) Free energy simulation of AT-case including all interactions. (**B**) Simulations omitting thymine-methyl interactions with the C2′ sugar atom (and connected hydrogens) of the 5′-neighboring nucleotide. (**C**) Snapshot of a BI conformation. (**D**) Normalized density plot of sampled sugar pucker phase of the 3′ ring versus ε–ζ coordinate (indicating low-phase preference in BI and high-phase preference for BII states). (**E**) Normalized density plot of sampled sugar puckering of 5′ ring versus ε–ζ coordinate (indicating high-phase preference in BI and low-phase preference for BII states). (**F**) Snapshot of BII conformation with arrows highlighting sugar pucker phase induced shifts.

In order to better understand the effect of the steric interactions between methyl group and 5′-sugar, we investigated its influence on individual base-pair steps. Note, that methyl–sugar clashes can only occur in base-pair steps with a thymine base at the 3′-position (e.g. in ApT but not in TpA steps). Interestingly, the population of BII states in ApT steps is even more stabilized through the omission of methyl–sugar clashes than expected from the free energy profiles obtained as averages over the central segments (compare Figure 3 with Figure 2). Intriguingly, the omission of methyl-5′-sugar interactions leads to a remarkable destabilization of BII states in the juxtaposed TpA steps. Based on the calculated free energies along the ε–ζ coordinate we conclude that methyl–sugar clashes destabilize BII conformations of ApT steps by ∼2 kcal/mol but at the same time the BII state of neighboring steps is stabilized (albeit to a lesser degree of ∼0.5 kcal/mol for TpA steps). This finding reflects the anti-correlation of DNA base-pair steps, a phenomenon which has also been subject of previous studies (18,36).

Our simulations suggest a qualitative sterical explanation of this nearest-neighbor anti-correlation that is illustrated in Figure 3C–F. For an ApT step in the BI configuration, the 3′ sugar pucker (T-nucleotide) adopts preferably a lower pucker phase than the 5′ sugar (A nucleotide) to adopt a well stacked configuration. A BII configuration, on the contrary, forces the 3′ sugar to adopt preferably a higher pucker phase that inclines the sugar ring in order to keep a near planar stacking geometry of adjacent bases (Figure 3). The opposite is observed for the 5′-sugar. Since this latter sugar adopts the role of a 3′ sugar in the consecutive step and it has been shifted to lower phase by the BII state, the population of another BII step at this neighboring step is suppressed.

**Figure 4.**
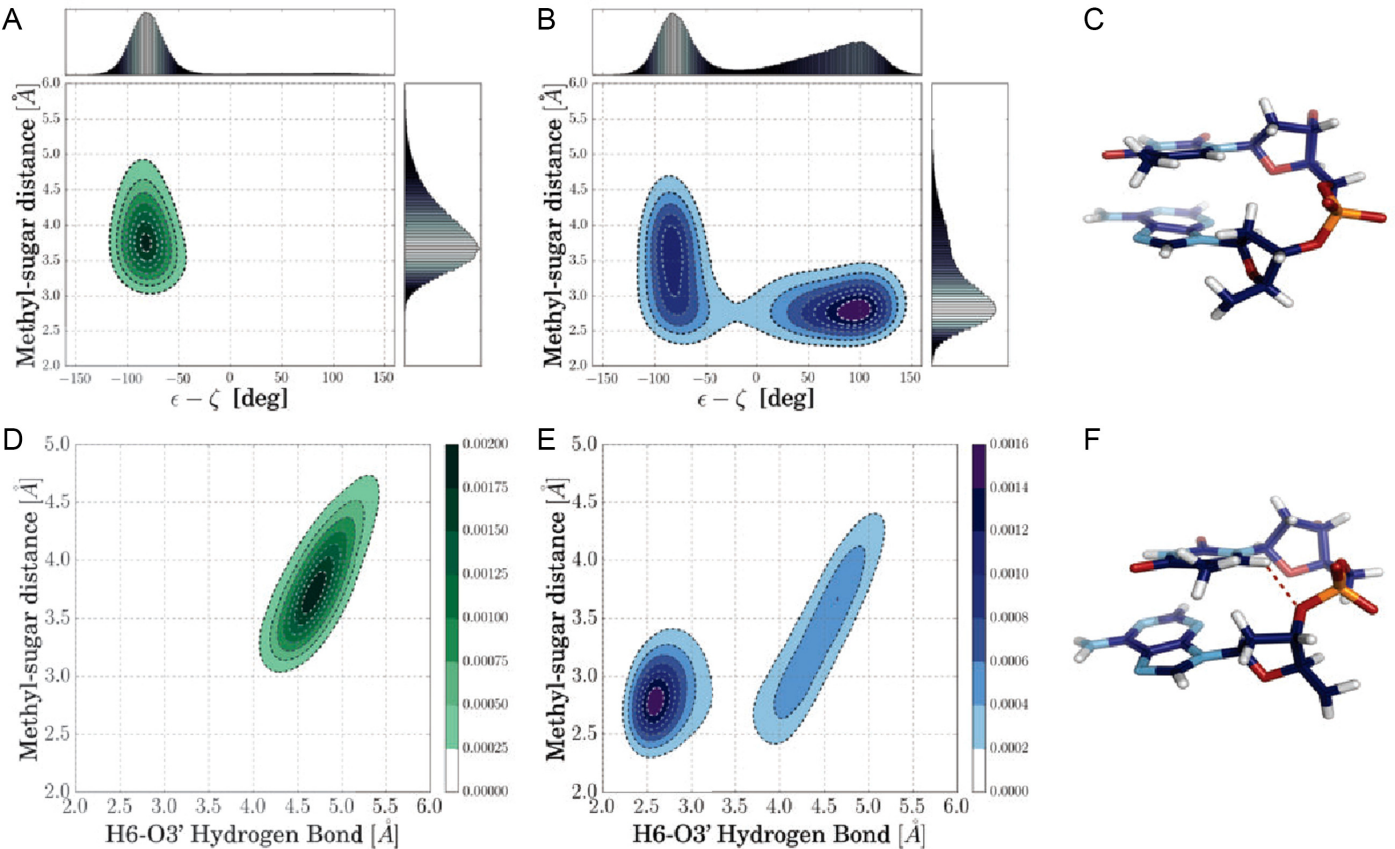
Methyl–sugar clashes destabilize the BII subspace in an ApT step. (**A**) Center of mass distance of thymine methyl group from C2′ atom of 5′ neighboring sugar versus ε−ζ coordinate in case of simulations including interactions of methyl groups with the C2′-atom of a 5′-sugar. BII states are populated very rarely. (**B**) Same as in (A) but switching-off interactions between methyl and sugar that allows for a closer approach of the groups. As a consequence, the BII subspace can also be accessed. (**C**) Snapshot of BI conformation, taken from simulation including all interactions. (**D**) Distance of thymine methyl group vs. H6-O3′ hydrogen bonding distance between thymine and 5′-sugar in case of simulations including all interactions (as in A). A close H6–O3′ hydrogen bonding distance between thymine and 5′-sugar is not formed under these conditions. (**E**) Same as in D but interactions between methyl and sugar are switched-off, giving rise to sampling of close H6–O3′ hydrogen bonding distances. (**F**) Snapshot of sampled BII conformation for simulation allowing clashes between methyl and sugar. The dashed red line indicates the unconventional hydrogen bond between thymine’s H6 and the backbone’s O3′ atom.

The simulations also indicate a qualitative sterical mechanism for the BII destabilization (by the methyl group) in case of the ApT steps as illustrated in Figure 4: The methyl-5′-sugar sterical interaction locks the bases to a specific conformational space in which the backbone preferentially adapts BI configurations. Switching off this sterical hindrance allows both components to come closer together whereby also the BII space becomes accessible (compare Figure 4A and B). The population of these states is then stabilized by unconventional hydrogen bonds between thymine’s H6-atom and the O3′ atom of the backbone sugar (Figure 4D and E). Notably, the existence and correlation to the BII population of these unconventional hydrogen bonds has already been pointed out by Balaceanu *et al.* (19), however, such hydrogen bonds are sterically possible only at specific base pair steps.

**Figure 5.**
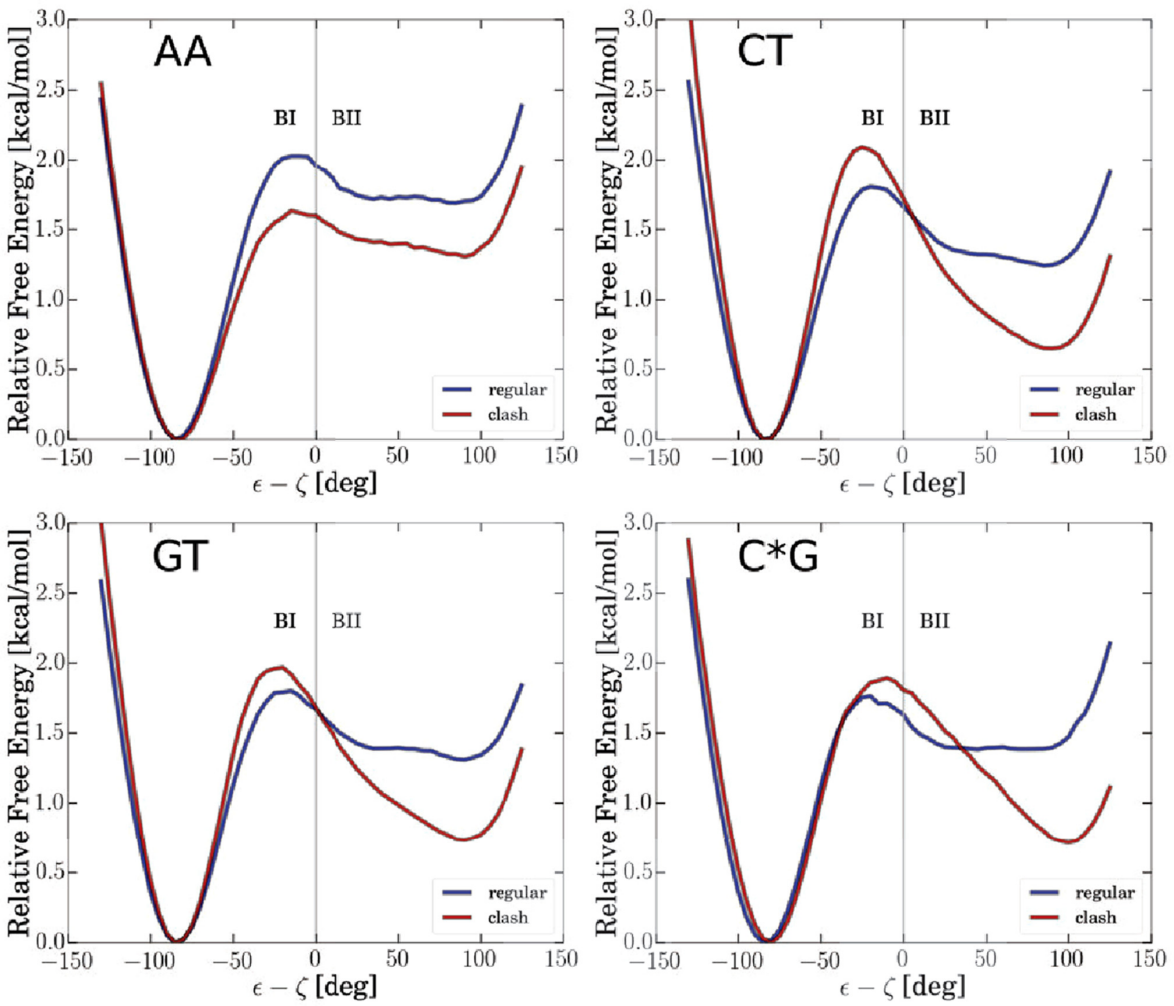
Calculated free energy profiles versus ε–ζ coordinate for the AA, CT, GT and C*G sequences (blue lines correspond to simulations including all interactions whereas red lines indicate simulation results omitting interactions of the base methyl group and the 5′ neighboring sugar C2′ atom and connected hydrogens).

In a next step, we studied the influence of methyl–sugar clashes for other sequences (Figure 5). Similar to the ApT steps (see above), we also find that methyl–sugar clashes are responsible for blocking H6-O3′ hydrogen bonds for GpT, CpT and GpC* steps (Supplementary Information Figures S12–S14). Note that we observed a different pattern for TpT steps, where H6-O3′ bonds are rare even when methyl–sugar interactions are switched off. We rather find that H6-O5′ hydrogen bonds are decisive for this sequence (Supplementary Information Figures S15 and S16). For simplicity, we considered the BI/BII population as average over the central DNA segment, though the same anti-correlation trend as before is obtained on the base-pair step level (illustrated in Supplementary Information Figures S17–S21). Similar to the comparison of the AT and AU cases we find that methyl-5′-sugar clashes destabilize BII states for each investigated sequence. Indeed, the CT, GT and C*G (methylated cytosine) cases show an even stronger increase in BII population upon omission of methyl–sugar interactions than the AT-sequence (Figure 5). The sequence dependence of DNA backbone substates has also been studied experimentally. Based on NMR experiments, it has been shown that out of the ten dinucleotide steps, the four steps which contain a thymine on the 3′ position (ApT, GpT, TpT and CpT) clearly exhibit the lowest BII population (2). This confirms our results that methyl groups (specifically at the 3′ position) destabilize BII backbone states due to methyl–sugar interactions.

### Methyl–sugar clashes influence DNA’s global structure and flexibility

The conformation of DNA’s backbone is strongly coupled to the configuration of the base-pairs (3,21,37,38). Indeed, the sterical methyl–sugar interactions also have an impact on the overall structure and flexibility of DNA in the present simulations. As relevant parameters we consider the mean twist, stretching and bending of the central DNA segments.

**Figure 6.**
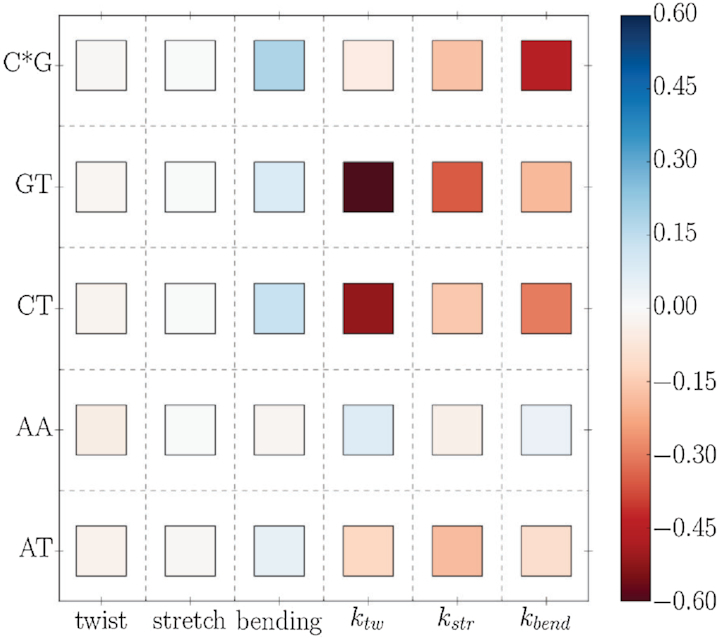
Relative changes in structure and flexibility due to methyl–sugar clashes. The first three columns indicate for each sequence the relative change in equilibrium twist, stretch and bending of the central segment whereas the last three columns represent the change in flexibility (indicated as the change in the calculated stiffness constant). For the latter cases, red entries mean that methyl sugar clashes have a decreasing/softening effect and blue entries represent an increase of the stiffness. All changes are given as relative to the reference case of reassignment of thymines’ and methylated cytosines’ bases, i.e. the entries reveal the influence of the van der Waals interactions between methyl and sugar group.

Whereas changes in the equilibrium twist and stretching are negligible, the intrinsic bending of the DNA double helix is markedly increased by methyl–sugar repulsion for most sequences (Figure 6), e.g. for the methylated cytosine sequence (C*G) an increase by ∼18% was observed. We calculated the DNA stiffnesses based on a harmonic stiffness model (assuming an underlying quadratic free energy surface for twisting, bending and stretching) that has also been used in previous studies (5,6,39–43),

(3) \begin{equation*} {K} = k_{{\rm B}} T \cdot \mathrm{C^{-1}}, \end{equation*}

where *k*_B_ and *T* indicate Boltzmann constant and temperature, respectively. *K* denotes the stiffness- and C the covariance-matrix of helical parameter fluctuations recorded during simulations (see Supplementary Information Section 5). The stiffness parameters were calculated with respect to the averages of the central four base pair steps. It is important to note, that the harmonic stiffness model is fully valid on this scale, as the steps superpose to single Gaussian distributions (indicating a quadratic underlying effective free energy profile) whilst individual steps can show clear bimodal behavior (Supplementary Information Figure S22).

Methyl–sugar repulsion is found to cause significant decreases in twist-, stretch- and bending-stiffness making the DNA-molecule overall more flexible. methyl–sugar repulsion alone significantly decreases the bending-stiffness by ∼40% for the methylated cytosine sequence (C*G) and by ∼20–30% for the GT and CT sequence. The largest changes are found for the twist-stiffness of the CT and GT sequence, where it has been found that methyl–sugar clashes soften by ∼50–60%. Besides, also the stretching flexibility is enhanced through methyl–sugar clashes, with the GT sequence showing the largest effect (∼35%).

It should be emphasized that these findings do not necessarily indicate that DNA molecules containing methylated cytosines are more flexible than sequences with unmethylated cytosine since we only evaluate the influence of methyl–sugar interactions by switching-off interactions between methyl and sugar group. It emphasizes that DNA’s local and global deformability is strongly influenced by these interactions. However, van-der-Waals interactions between methyl groups and other chemical groups are still included. Previous studies have pointed out that methylated cytosine sequences are overall stiffer than their regular analogs (43). Based on comparative simulations (Supplementary Information Section 7) we obtained the following trend: On the level of base pair steps, GpC* steps are significantly stiffer than GpC steps, while C*pG steps are overall more flexible than CpG steps. On the level of the whole central segment stretching and bending stiffnesses are slightly lower for the methylated case (C*G case) compared to the CG case (∼5–7%), but the twisting-stiffness of the C*G case is significantly higher compared to the unmethylated central CG sequence (∼20%, Supplementary Information Tables S1-S3). We also checked in how far the charge-reassignment alone of methylated-bases influenced changes in DNA’s structure and flexibility (Supplementary Information Figures S23–S24). Here, our simulations indicate, that such charge effects are relevant for methylated cytosine sequences, but are negligible for canonical sequences.

**Figure 7.**
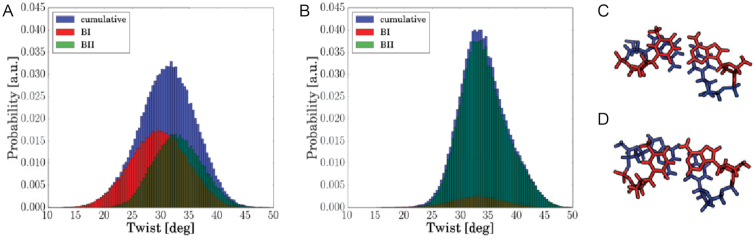
Methyl sugar clashes increase DNA flexibility at the central segment. (**A**) Calculated twist distribution of a GpT step in case of simulations including methyl sugar interactions indicating the total cumulative distribution (both sampled BI and BII states) as well as distributions considering BI and BII states separately. The distribution at the BI states is shifted and broader (standard deviation σ = 7.2°) than that for BII states (σ = 6.2°). The standard deviation of the cumulative distribution amounts to σ = 7.1°. (**B**) Twist distribution upon removing methyl–sugar interactions: The cumulative twist distribution is narrower since the population of BII is strongly increased (σ = 5.7°). (C) Snapshot of undertwisted GpT step (∼20°) at ε–ζ ≃ −80° in Watson and Crick strand. (**D**) Snapshot of overtwisted GpT step (∼38.5°) at ε–ζ ≃ −80° in both strands.

The effect of methyl–sugar clashes to increase flexibility is counterintuitive (restriction to BI substate), however, this is a direct consequence of the influence of the backbone: The twist-distribution within the BI states is broader than that of BII states (Figure 7A). Thus, an increased population of BII states results in a narrower overall twist distribution (Figure 7B). The lower variance in twist directly reflects a higher stiffness with respect to this mode. Note, that from the ε–ζ free energy profiles one would expect that the twist-distribution of BI states is narrower, however, the backbone population does not map linearly to the twist variable. Indeed, similar ε–ζ configurations can show different twisting (illustrated in Figure 7C and D).

## CONCLUSIONS

The methyl group in thymine and in C5-methylated cytosine modulates the BI-BII substate distribution and the DNA flexibility. Since DNA methylation plays a key role in epigenetic regulation of gene expression (44–46), it is likely that its influence on DNA deformability is also linked to its biological function (43,44,47,48). In the presented study, we addressed the correlation between methyl groups and BI/BII promiscuity using comparative MD simulations. As a key technique we employed sets of simulations that specifically included or omitted non-bonded interactions between methyl groups and hypothetically important partner groups. Based on these simulations, we showed that the hydrophobicity of methyl groups as well as methyl-π stacking that had been proposed to cause changes in the BI/BII ratio (24,25,27) exhibit only a small influence on the population of BI/BII substates. Switching-off interactions between methyl group and the C2′ atom and its hydrogen atoms, in contrast, stabilized BII and hence decreased population of BI conformations significantly. This trend confirms results of previous studies (23,26,28) and was found for each investigated sequence, albeit to different degrees: The strongest changes appeared for sequences including CpT and GpT steps or methylated cytosines. Previous studies indicate a decisive role of the formation of unconventional base-sugar H6–O3′ hydrogen bonds (or H8–O3′ in case of purines) for stabilizing the BII state (19–21) that is also found in the present simulations. However, since such bonds are in principle possible for every dinucleotide step the correlation alone does not explain the physical reason for the BI/BII sequence dependence. Based on sterical considerations it was possible to qualitatively explain the effect of the methyl group on the BI/BII ratio. Interestingly, an increased propensity of BII at one step results in a reduced BII probability at a neighboring step that can be qualitatively explained by a coupling to the nearest-neighbor sugar pucker conformation. Given the pronounced impact of the interaction between methyl and sugar group it also influences the global structure and flexibility of DNA (29–31). While these interactions increase the intrinsic DNA bending, a decrease in the DNA’s stiffness was observed. For CpT and GpT rich sequences, the steric clashing between methyl and sugar group decreases DNA’s torsional rigidity by up to ∼60%. We found as a main sterical reason that the twist distribution of states within the BI basin is broader than in case of sampled states within the BII regime. Consequently, since the methyl-5′-sugar interactions stabilize the BI state the twist flexibility increases. The comparative simulation methodology of including or omitting specific non-bonded interactions could be applied in future studies on other phenomena like phosphate repulsion or the role of hydrogen bonding in structure formation could be tackled.

## Supplementary Material

Supplementary Data
