# Supplementary material for: How methyl–sugar interactions determine DNA structure and flexibility
Source: Nucleic Acids Res. 2018 Dec 12;47(3):1132–40. doi: 10.1093/nar/gky1237 (PMC6379717; doi:10.1093/nar/gky1237)
Supplement: Supplementary Data [file gky1237_supplemental_files.pdf]

Supplementary Information

How methyl-sugar interactions determine DNA structure and flexibility

Korbinian Liebl and Martin Zacharias

November 23, 2018

Contents

1 Calculation of $\epsilon - \zeta$ Free Energy Profiles 3

2 Calculation of standard errors 3

3 Convergence of the MD trajectories 3

4 Patterns of unconventional Hydrogen-Bonding 4

5 Calculation of helical parameters and flexibilities 4

6 Influence of charge reassignment 5

7 Comparison between cytosine and methylated-cytosine 5

List of Figures

S1 Calculated free energy profiles for AT/AU-sequences including error bars 7
S2 Convergence of calculated free energy profile for AT-sequence 8
S3 Convergence of calculated free energy profile for AA-sequence 9
S4 Convergence of calculated free energy profile for CT-sequence 10
S5 Convergence of calculated free energy profile for GT-sequence 11
S6 Convergence of calculated free energy profile for C*G-sequence 12
S7 Calibration AT 12
S8 Calibration AA 13
S9 Calibration CT 13
S10 Calibration GT 14
S11 Calibration C*G 14
S12 H6-O3' hydrogen bonding vs. methyl-sugar distance, CpT-case 15
S13 H6-O3' hydrogen bonding vs. methyl-sugar distance, GpT-case 15
S14 H6-O3' hydrogen bonding vs. methyl-sugar distance, GpC*-case 16
S15 H6-O3' hydrogen bonding vs. methyl-sugar distance, ApA-case 17
S16 H6-O5' hydrogen bonding vs. methyl-sugar distance, TpT-case 17
S17 Backbone Correlation of AT-sequence 18
S18 Backbone Correlation of AA-sequence 18
S19 Backbone Correlation of CT-sequence 19
S20 Backbone Correlation of GT-sequence 19
S21 Backbone Correlation of C*G-sequence 20
S22 Comparison of twist probability steps at single steps vs. average over 4 base pair steps 21
S23 Influence of charge reassignement on DNA's structure and flexibility . . 22
S24 Comparison of structure and flexibility between regular and clash-omitting simulations 22

1 Calculation of $\epsilon - \zeta$ Free Energy Profiles

From the trajectories obtained during data gathering we determined the sampled ϵ and ζ dihedral angles using Curves+ [1] of the central four base-pair-steps of both Watson and Crick strands. Relative free energy profiles along $\epsilon - \zeta$ were calculated by dividing the $\epsilon - \zeta$ coordinate in bins of 5°. For each bin, we counted the number of samples. The probability for each bin was determined as ratio of sampled states in the bin vs. total number of samples. The free energy of each bin was obtained by Boltzmann-Inversion:

$$\mathrm{F} = -\mathrm{k_B T} \cdot \ln(\mathrm{p}) \tag{1}$$

From each bin, we subtracted the value of the global free energy minimum, thus obtaining a relative free energy profile along $\epsilon - \zeta$.

2 Calculation of standard errors

We calculated the standard errors of each bin's free energy as

$$\Delta\mathrm{F} = \frac{-\mathrm{k_B T}}{\mathrm{p}} \cdot \Delta\mathrm{p}, \tag{2}$$

where Δp denotes the standard error in a bin's probability. We determined Δp by:

$$\Delta\mathrm{p} = \frac{1}{N}\sqrt{N_{bin} \cdot (1-p)^2 + (N - N_{bin}) \cdot p^2} \tag{3}$$

N is the total number of samples, N_{bin} the number of sampled states in the bin and p denotes the probability of the bin. Free energy profiles along $\epsilon - \zeta$ including error bars are illustrated in Fig. S1.

3 Convergence of the MD trajectories

Convergence of the MD simulation results were checked by splitting the trajectories into subintervals, and generating a free energy profile along $\epsilon - \zeta$ for each subinterval. Trajectories were split into intervals of $1\%, 10\%, 75\%$ and 100% of the total simulation times. The calculated free energy profiles along the $\epsilon - \zeta$ coordinate for each subinterval are illustrated in Fig. S2,S3,S4,S5,S6 We obtained the trend, that already only 10% of the simulations reproduce results from the complete simulation quite precisely, indicating convergence of the BI/BII distribution in the MD simulations.

For the sequences AA, CT, GT and C*G we find that omission of methyl-π stacking destabilizes the BII subspace, i.e. methyl-π stacking increases the conversion from BI to BII.

4 Patterns of unconventional Hydrogen-Bonding

For each sequence, we analyzed the contacts between the pyrimidine H6 and the 5'-neighboring sugar O3' atom. This contact is frequently considered as unconventional H6-O3' hydrogen bonding. Methyl-sugar clashes suppress the BII state, which is in general stabilized by unconventional H6-O3' bonds. Simulations during which methyl-sugar clashes were allowed record strong H6-O3' interactions, except for the AA sequence, which exhibits significant H6-O5' hydrogen bonds. This qualitative, sterical difference explains why the deformability of A-tracts is less influenced by methyl-sugar clashes.

5 Calculation of helical parameters and flexibilities

Helical parameters were calculated with Curves+ [1]. In this study, we consider the parameters twist, stretch and bending. Stretch terms were calculated by the mean value of the helical rise of each base-pair step. Bending terms were calculated as the curvature term introduced by Strahs and Schlick [2]:

$$\text{curvature} = \sqrt{\theta_{\mathrm{T}}^2 + \theta_{\mathrm{R}}^2}, \quad \text{with} \tag{4}$$

$$\theta_T = \sum_{j=N_s}^{N_f} \tau_j \cos\left(\sum_{i=Ns}^{j} t_i\right) + \rho_j \sin\left(\sum_{i=N_s}^{j} t_i\right) \tag{5}$$

$$\text{and} \tag{6}$$

$$\theta_R = \sum_{j=N_s}^{N_f} -\tau_j \sin\left(\sum_{i=Ns}^{j} t_i\right) + \rho_j \cos\left(\sum_{i=N_s}^{j} t_i\right) \tag{7}$$

N_s and N_f denote the starting base-pair step and the final base-pair step. Structural parameters and flexibilities were evaluated only for the central four base pair steps. Thus, $\mathrm{N_s}$=6 and $\mathrm{N_f}$ = 9. τ_{j}, ρ_{j} and $\mathrm{t_i}$ denote the base-pair step parameters tilt, roll (of step j) and twist (of step i).
Stiffness parameters, given by a matrix K, can be obtained from the covariance matrix C:

$$\mathrm{K} = k_B T \cdot \mathrm{C}^{-1}$$

We calculated the stiffness of each parameter directly from the inverse of its variance, i.e. we did not set up a covariance matrix from which also coupling terms can be obtained. In this way, we determined one stiffness constant for each of the three parameters (twist, stretch, bending). These constants describe the stiffness of a parameter under the condition that all physically coupled parameters are unconstrained (effective stiffness).
We then calculated stiffness constants separately from simulations under the control of regular and modified parameter topologies, and evaluated relative differences between

constants obtained from regular and modified simulations.
It is important to note that our analysis procedure assumes Gaussian-distribution of the parameters (harmonic stiffness model). It is well established that individual base pair step-parameters can exhibit clearly non-single-Gaussian behavior in particular of the twist coordinate. However, mainly due to nearest-neighbor anti-correlation the distributions of multiple base-pair-steps superpose to single-Gaussian distributions. Figure S22 illustrates the twist-distribution of central, single base-pair-steps, which can significantly deviate from single Gaussian distributions. However, the twist over the central segment of 4 base pair steps (as mean per base pair step twist) shows clear Gaussian behavior and justifies an underlying quadratic effective free energy surface.

6 Influence of charge reassignment

Switching-off non-bonded interactions between methyl and a hypothetical important partner group also requires reassigning the charges of thymine or methylated cytosine. In order to determine the influence of this change in the electrostatic energy landscape, we performed simulations in which only the charges have been reassigned in accordance with the demethylated analog. Overall, we see that the differential electrostatic energy landscape causes only minor deviations in the population of backbone substates and DNA's global structure and flexibility. However, methylated cytosine exhibits distinctive features, as the charge reassignment makes the molecule softer with respect to the twist stiffness (Fig. S23). This result indicates that the increased torsional (twist) stiffness of methylated cytosine containing DNA compared to regular cytosine containing sequences is also influenced by electrostatic contributions.

7 Comparison between cytosine and methylated-cytosine

Based on the harmonic stiffness model, we also compared the stiffnesses of the methylated cytosine rich sequence (case C*G) with its unmethylated analog (CG) and checked in how far these differences are influenced by sugar methyl van der Waals interactions and the partial charges on the methyl group (see tables S1 to S3). Slightly lower stretching and bending stiffness parameters are found for the complete central segment for C*G compared to CG, whereas the global twisting is much stiffer for the C*G case (table S1). Besides the global deformability of the complete central segment, we also calculated local stiffness constants for the base pair steps. The simulations indicate that GpC steps are significantly softer than G*pC steps and vice versa for CpG steps (tables S2 and S3).

Table S1: Global stiffness parameters for CG and C*G sequence.

	twist $[\frac{kcal}{mol \cdot deg^2}]$	stretch $[\frac{kcal}{mol \cdot \AA^2}]$	curvature $[\frac{kcal}{mol \cdot deg^2}]$
CG	0.0091	3.4	0.0095
C*G	0.011	3.2	0.0091
C^*G^{clash}	0.0095	3.6	0.013

C* denotes C5-methylated cytosine. C^*G^{clash} denotes the simulation in which we allowed methyl-sugar clashing (and hence also accounts for charge redistribution).

Table S2: Elastic constants of GpC and GpC* steps, respectively.

	twist $[\frac{kcal}{mol \cdot deg^2}]$	roll $[\frac{kcal}{mol \cdot deg^2}]$	tilt $[\frac{kcal}{mol \cdot deg^2}]$	rise $[\frac{kcal}{mol \cdot \AA^2}]$	slide $[\frac{kcal}{mol \cdot \AA^2}]$	shift $[\frac{kcal}{mol \cdot \AA^2}]$
GpC	0.020	0.018	0.027	8.0	2.6	0.65
GpC*	0.024	0.025	0.047	7.1	2.6	1.8
GpC^{*clash}	0.035	0.026	0.047	8.4	3.1	1.4

C* denotes C5-methylated cytosine. GpC^{*clash} denotes the simulation in which we allowed methyl-sugar clashing (and hence also accounts for charge redistribution).

Table S3: Elastic constants of CpG and C*pG steps, respectively.

	twist $[\frac{kcal}{mol \cdot deg^2}]$	roll $[\frac{kcal}{mol \cdot deg^2}]$	tilt $[\frac{kcal}{mol \cdot deg^2}]$	rise $[\frac{kcal}{mol \cdot \AA^2}]$	slide $[\frac{kcal}{mol \cdot \AA^2}]$	shift $[\frac{kcal}{mol \cdot \AA^2}]$
CpG	0.012	0.015	0.021	5.2	2.1	0.76
C*pG	0.018	0.013	0.016	5.7	1.7	0.58
C^*pG^{clash}	0.012	0.017	0.029	5.4	2.6	1.1

C* denotes C5-methylated cytosine. C^*pG^{clash} denotes the simulation in which allow methyl-sugar clashing (and hence also accounts for charge redistribution).

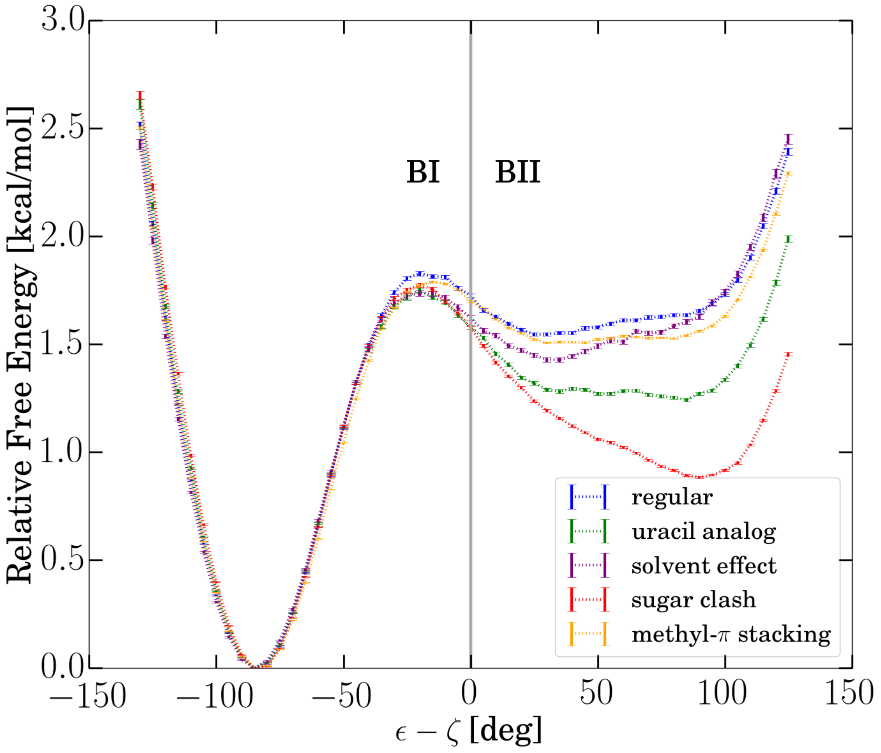

Figure S1: Calculated free energy profiles along the $\epsilon - \zeta$ coordinate for DNA simulations with AT/AU-sequences including error bars. Relative free energies were calculated by Boltzmann inversion of the sampled probability distributions.

References

[1] Lavery, R., Moakher, M., Maddocks, J., Petkeviciute, D., and Zakrzewska, K. (2009) Conformational analysis of nucleic acids revisited: Curves+. *Nucleic Acids Res.,* **37**(17), 5917–5929.

[2] Strahs, D. and Schlick, T. (2000) A-Tract Bending: Insights into Experimental Structures by Computational Models. *J. Mol. Biol.,* **301**(3), 643–663.

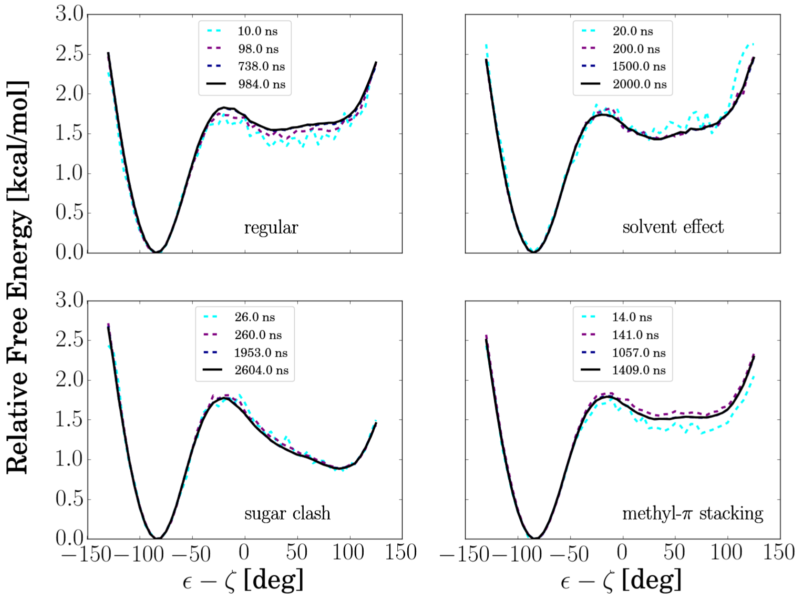

Figure S2: Calculation of free energy profiles along the $\epsilon - \zeta$ coordinate from simulations of different lengths (simulation times and line colors given in inset) for the AT-sequence. Simulations under the control of four force field variants are considered. 'Regular': Simulation under standard force-field conditions. 'solvent effect': All non-bonded interactions between central methyl groups and water molecules were switched-off. 'sugar clash': Non-bonded interactions between methyl-groups and the C2' atom (and its hydrogens) of the 5' neighbored base were switched-off. 'methyl-π stacking': Non-bonded interactions between methyl-groups and all atoms of the 5' neighbored base were switched-off.

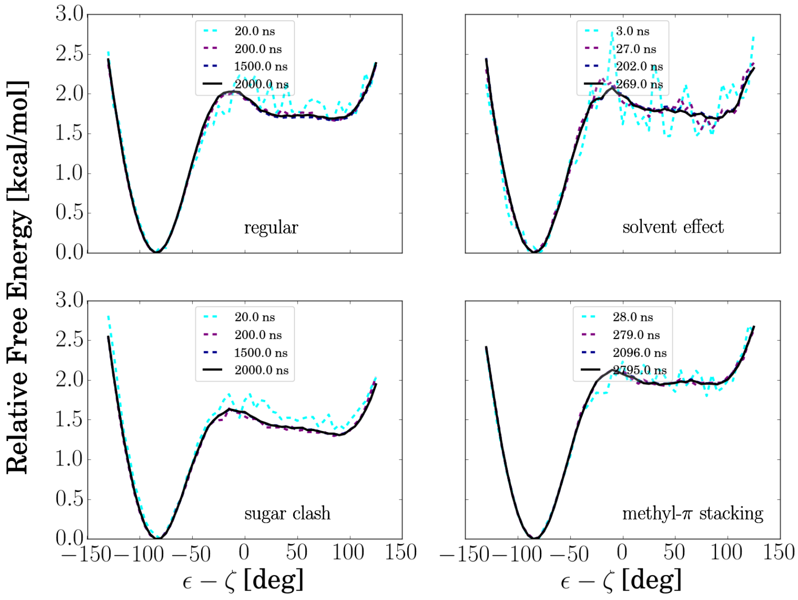

Figure S3: Same as legend of Fig. S2 but for the AA-case.

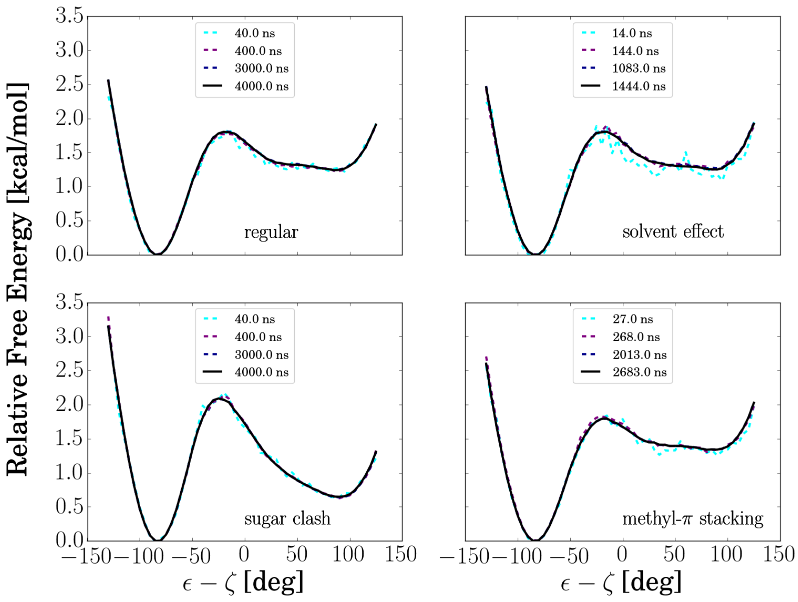

Figure S4: Same as legend of Fig. S2 but for the CT-case.

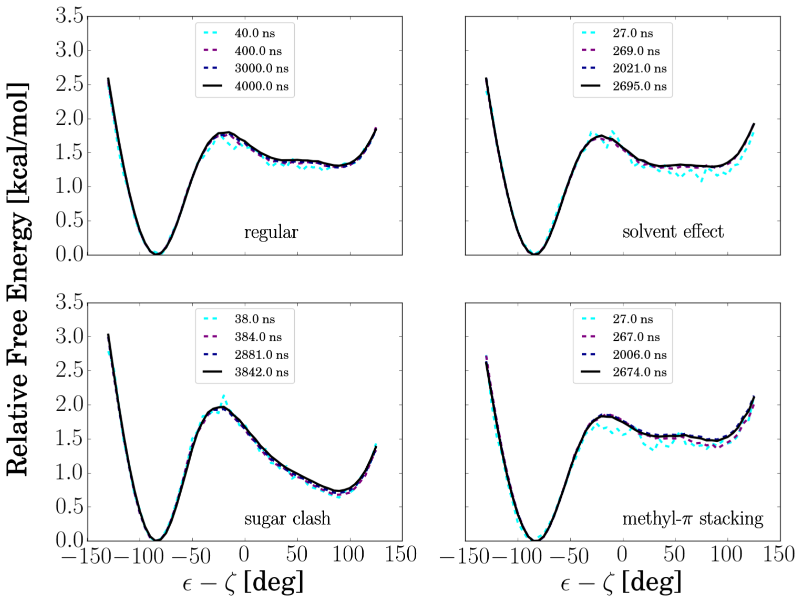

Figure S5: Same as legend Fig. S2 but for the GT-case.

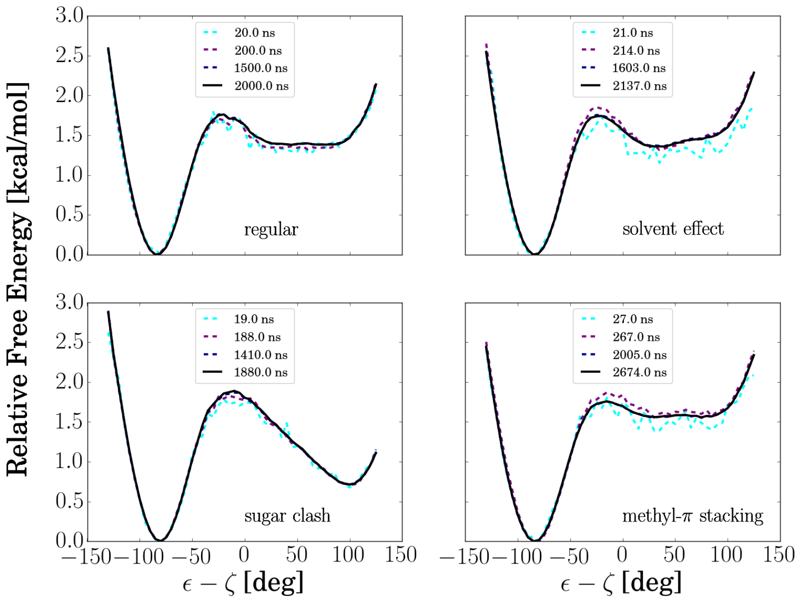

Figure S6: Same as legend Fig. S2 but for the C*G-case.

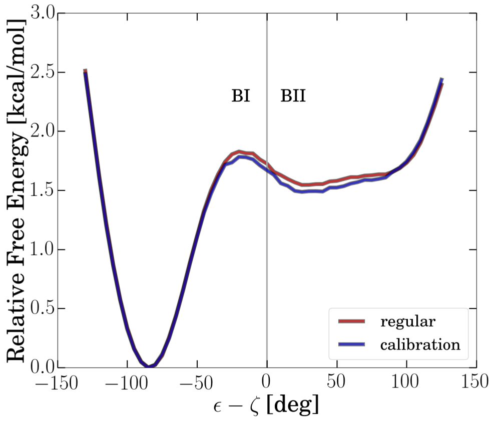

Figure S7: Influence of charge redistribution (after removing partial charges on the thymine methyl group and redistribution of charges to represent the demethylated base) for AT-sequence: Free energy profiles along $\epsilon - \zeta$ for regular case (standard force field) and charge re-distribution (calibration) of thymine bases (no partial charges on methyl group).

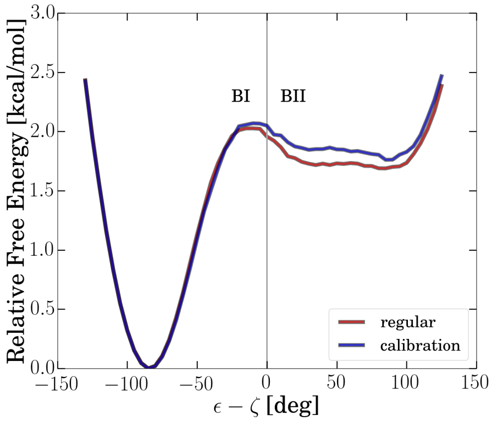

Figure S8: Influence of charge redistribution for AA-sequence: Free energy profiles along $\epsilon - \zeta$ for regular case (standard force field) and charge reassignment (calibration) of thymine bases (no partial charges on methyl group).

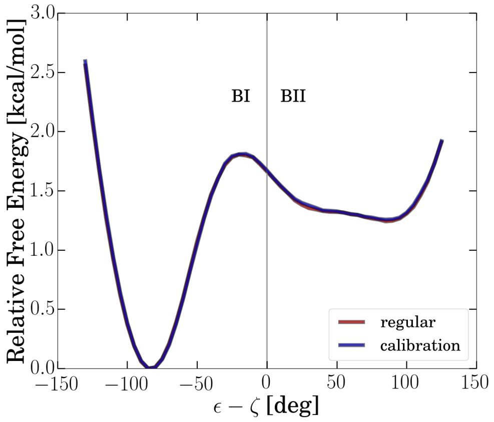

Figure S9: Influence of charge redistribution for CT-sequence: Free energy profiles along $\epsilon - \zeta$ for regular case (standard force field) and charge reassignment (calibration) of thymine bases (no partial charges on methyl group).

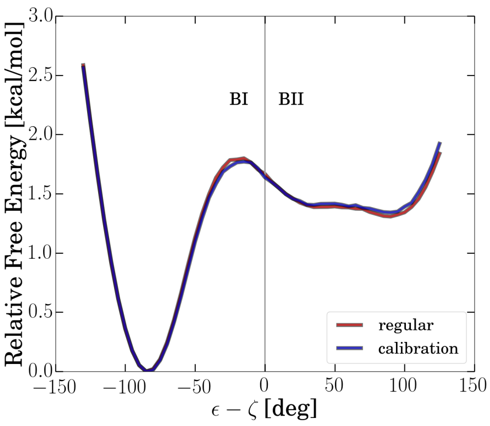

Figure S10: Influence of charge redistribution for GT-sequence: Free energy profiles along $\epsilon - \zeta$ for regular case (standard force field) and charge reassignment (calibration) of thymine bases (no partial charges on methyl group).

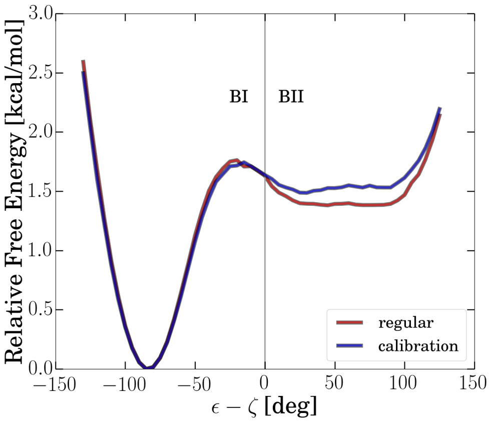

Figure S11: Influence of charge redistribution for C*G-sequence: Free energy profiles along $\epsilon - \zeta$ for regular case (standard force field) and charge reassignment (calibration) of methylated cytosine bases (no partial charges on methyl group).

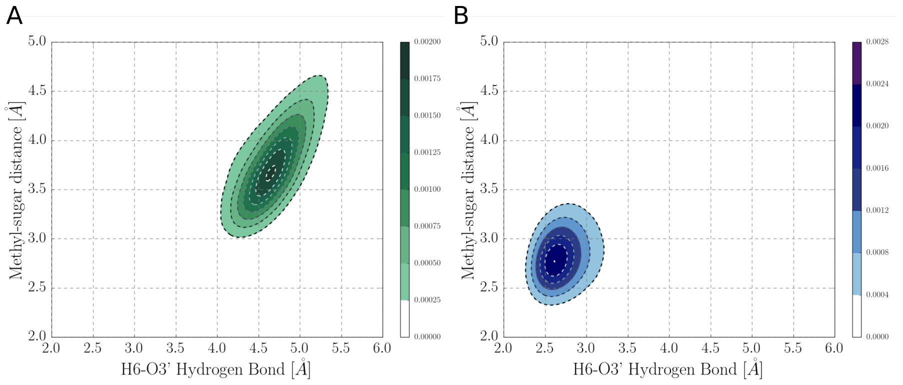

Figure S12: Hydrogen-Bonding pattern for CpT steps (plotted as normalized sampling density): A) Under regular (standard force field) conditions, unconventional H6-O3' hydrogen bonds are suppressed. B) If the methyl-sugar clash is allowed, CpT steps occupy a conformational space, which is characterized by H6-O3' hydrogen bonds mediated through the approaching methyl and sugar group.

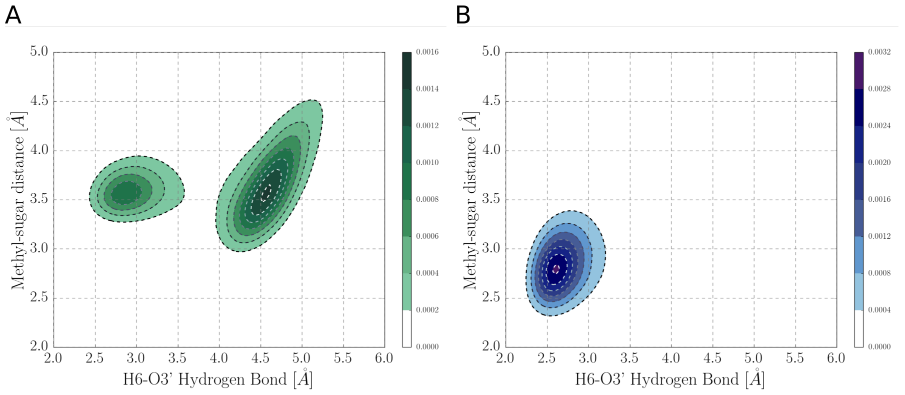

Figure S13: Hydrogen-Bonding pattern for GpT steps (plotted as normalized sampling density): A) Under regular conditions (standard force field), unconventional H6-O3' hydrogen bonds are suppressed. B) Releasing methyl-sugar clashes allows GpT steps to occupy a conformational space, which is characterized by H6-O3' hydrogen bonds mediated through the approaching methyl and sugar group.

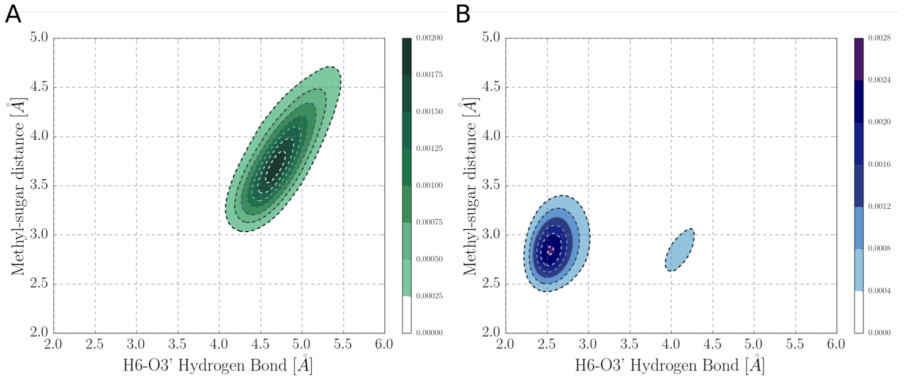

Figure S14: Hydrogen-Bonding pattern for GpC* steps, with C* denoting C5-methylated cytosine, shows same behavior than thymine-rich sequences (plotted as normalized sampling density): A) Under regular (standard force field) conditions, methyl and sugar group cannot approach each other, hence H6-O3' hydrogen bonds are suppressed. B) Allowing methyl-sugar clashes results in pronounced hydrogen-bonding between the H6 and O3' atom.

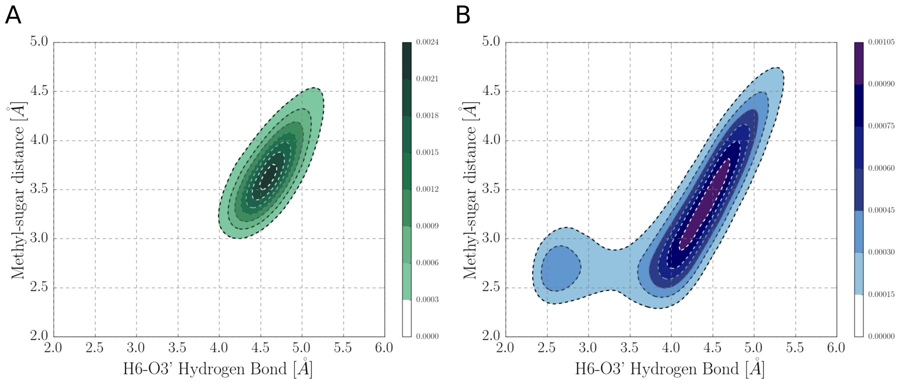

Figure S15: AA-sequence contains hydrogen-bonding patterns for TpT steps (plotted as normalized sampling density). A) 2d-Histogram of H6-O3' distance and distance between methyl and sugar group obtained from regular simulation. Methyl and sugar group avoid sterical clashes and hence keep a distance of at least $3\AA$. In such conformations, H6-O3' hydrogen bonds are sterically not possible. B) 2d-Histogram of H6-O3' distance and distance between methyl and sugar group obtained from simulations, during which methyl-sugar interactions were switched-off. Here, methyl and sugar group are allowed to approach each other and consequently also H6-O3' hydrogen bonds can be formed, albeit rarely. Thereby, the A-tract sequence reacts fundamentally different upon allowing methyl-sugar clash compared to other sequences. For the latter, H6-O3' bonding is a dominant feature.

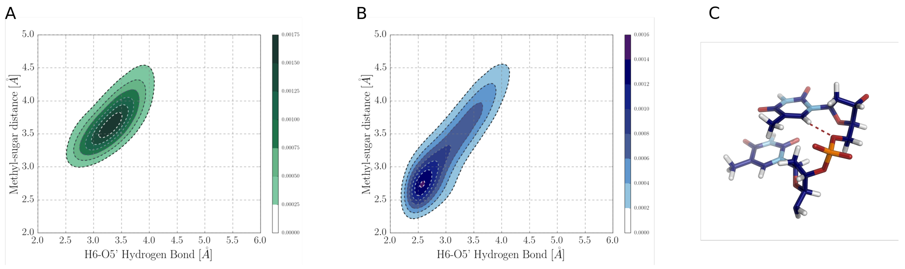

Figure S16: Hydrogen-Bonding pattern for TpT steps: A) H6-O5' hydrogen bonds are naturally rare due to methyl-sugar clashing. B) If methyl sugar interactions are switched-off, H6-O5' (rather than H6-O3') hydrogen bonds are a central motif. C) Snapshot of a TpT step, the dashed red line indicates the unconventional hydrogen bond between thymine's H6 atom and the O5' atom of the backbone.

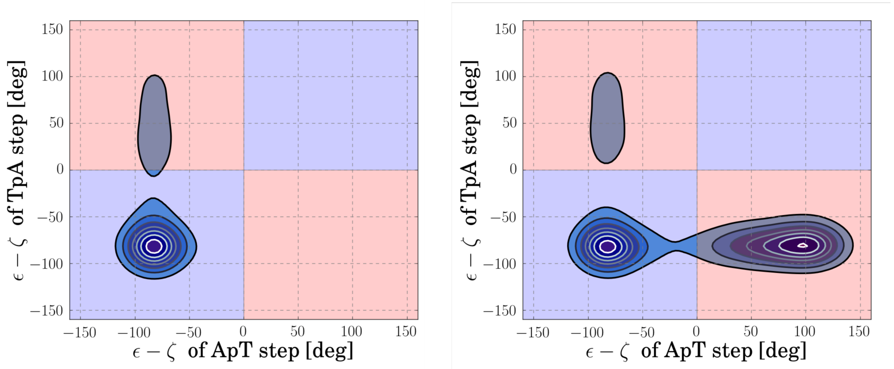

Figure S17: AT-sequence: $\epsilon - \zeta$ distribution (as normalized sampling density) of ApT and neighbored TpA steps for regular case (left) and omission of methyl-sugar interactions (right). Subspaces which indicate correlation between the adjacent steps are marked in blue, subspaces of anti-correlation are in red.

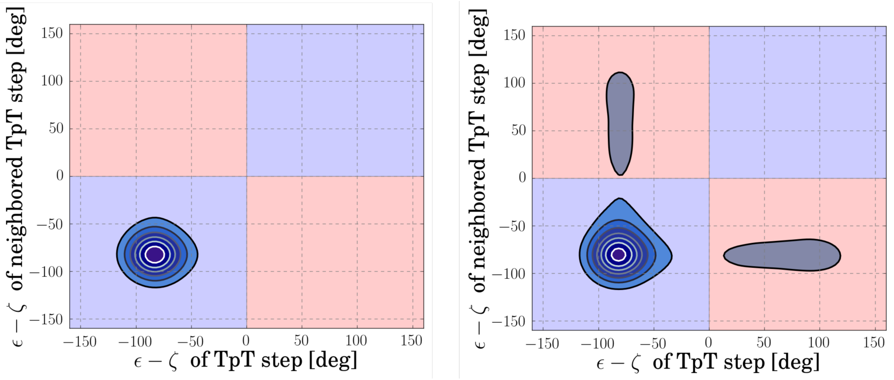

Figure S18: AA-sequence: $\epsilon - \zeta$ distribution (as normalized sampling density) of a TpT versus its neighbored step for regular case (left) and omission of methyl-sugar interactions (right). Subspaces which indicate correlation between the adjacent steps are marked in blue, subspaces of anti-correlation in red.

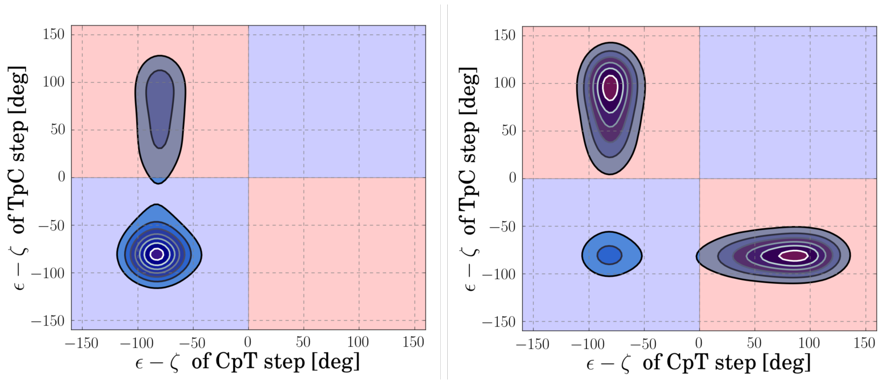

Figure S19: CT-sequence: $\epsilon - \zeta$ distribution (as normalized sampling density) of CpT and neighbored TpC steps for regular case (left) and omission of methyl-sugar interactions (right). Subspaces which indicate correlation between the adjacent steps are marked in blue, subspaces of anti-correlation in red.

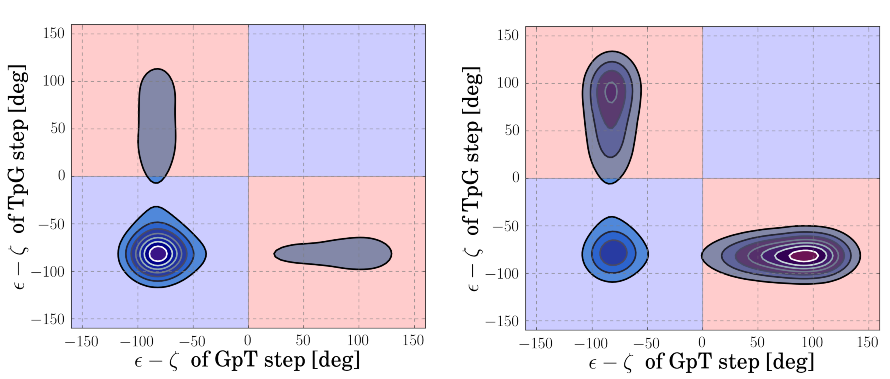

Figure S20: GT-sequence: $\epsilon - \zeta$ distribution (as normalized sampling density) of GpT and neighbored TpG steps for regular case (left) and omission of methyl-sugar interactions (right). Subspaces which indicate correlation between the adjacent steps are marked in blue, subspaces of anti-correlation in red.

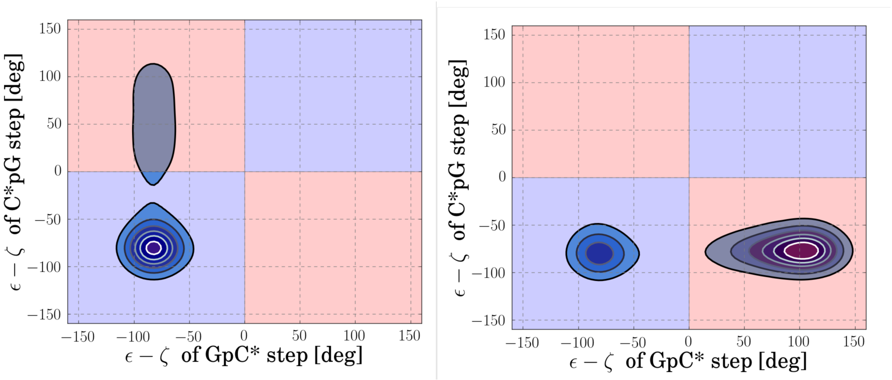

Figure S21: C*G-sequence: $\epsilon - \zeta$ distribution (as normalized sampling density) of GpC* and neighbored C*pG steps for regular case (left) and omission of methyl-sugar interactions (right). Subspaces which indicate correlation between the adjacent steps are marked in blue, subspaces of anti-correlation in red.

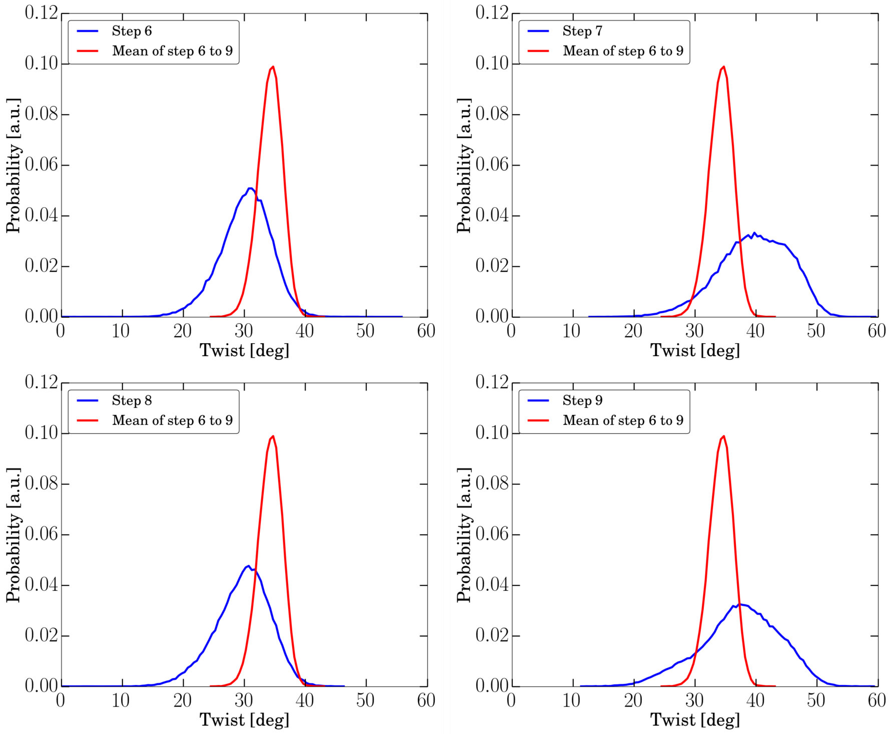

Figure S22: Probability distribution of a single base pair step's twist (blue curves) reveals non-unimodal (non-Gaussian) behavior. The mean over multiple base-pair steps superposes to a Gaussian (red curves). These schematic plots were recorded from the AT sequence with regular topology. The plots were generated by dividing the twist coordinate into bins of 0.5°, the probability of each bin is then given by the ratio of bin-samples to total number of samples.

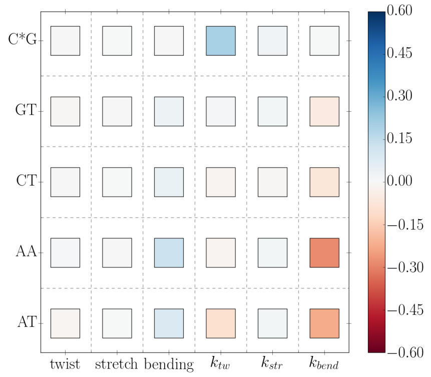

Figure S23: Influence of charge reassignement of thymine and methylated cytosines on DNA's structure and flexibility. Red entries mean that charge reassignement (no partial charges on methyl groups but van Der Waals interactions included) has an increasing/stiffening effect. Blue entries illustrate decreasing/softening entries.

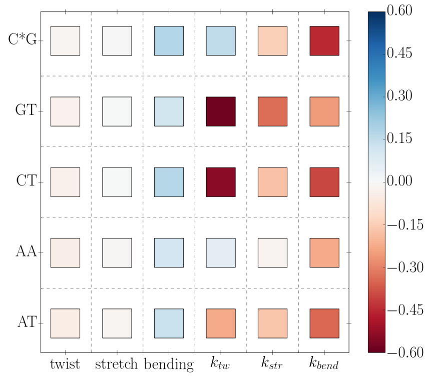

Figure S24: Comparison of structure and flexibility between regular and clash-omitting simulations. Relative changes of parameters obtained from regular trajectories and from trajectories in which non-bonded interactions of methyl and sugar group have been omitted. The plot was generated in the same way as Fig S23.
